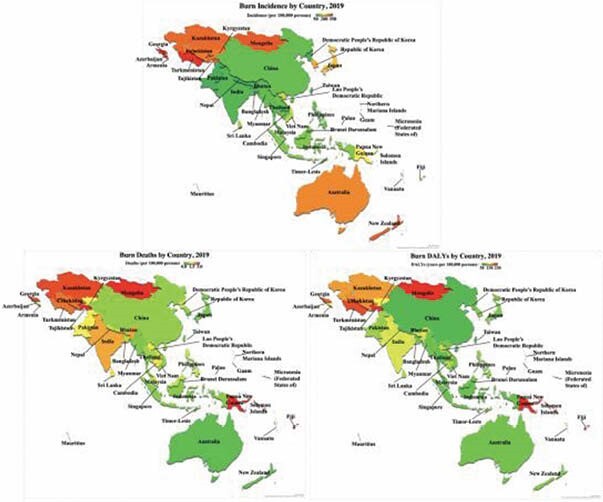# 58 Burn Injuries in Asia: A Global Burden of Disease Study

**DOI:** 10.1093/jbcr/irac012.061

**Published:** 2022-03-23

**Authors:** Zachary J Collier, Katherine McCool, William P Magee, Tom Potokar, Justin Gillenwater

**Affiliations:** Keck School of Medicine, University of Southern California, Los Angeles, California; Keck School of Medicine, University of Southern California, Los Angeles, California; Children’s Hospital Los Angeles, Los Angeles, California; Interburns, Geneva, Geneve; USC/LAC+USC Medical Center, Los Angeles, California

## Abstract

**Introduction:**

Burn injuries disproportionately affect low- and middle-income countries. Work conditions, rapid industrialization, social conditions, cultural activities, political conflict, and lack of access to safe and affordable surgery are key barriers to effective burn care in Asia. This study aimed to better define the burn burden in Asia, its sub-regions, and related sex and age disparities to elucidate populations where targeted burn care interventions are most needed.

**Methods:**

The 2019 Global Burden of Disease (GBD19) of the Global Health Data Exchange was used to acquire 151,741 sources of epidemiological data on fire, heat, and hot substance-related injuries for 53 countries in Asia from 1990 to 2019. Measures used to derive summative statistics included incidence, deaths, disability-adjusted life years (DALYs), and mortality ratio (deaths: incidence) by year, sex, age, and location. Spatial mapping was performed to geographically delineate burn indicators.

**Results:**

From 1990 to 2019, an estimated 117 million burns occurred in Asia. The relative proportion of global burns, deaths, and DALYs from Asia increased during that time. By 2019, 46% of global burn cases, 47% of deaths, and 46% of DALYs were from Asia. The two most burdened regions were South and Southeast Asia, accounting for 30-40% of all global cases, deaths, and DALYs. Compared to global averages, the incidence, death, and DALY rates for Asia were 32%, 22%, and 23% higher. Central Asia had the worst rates, averaging 2.9, 2.3, and 2.6 times the global averages. Throughout Asia, men were 32%, 63%, and 47% more likely to be burned, die, and suffer DALYs than women versus the global disparities of 7%, 26%, and 10%. Only South Asia’s trend was reversed with women suffering 15%, 20%, and 27% more burns, deaths, and DALYs than men. In Asia, those under 5 years were most impacted by DALYs (314 years/100,000 people), 5-14 year olds had the highest burn rate (219 cases/100,000), and 70+ year olds had the highest death rate (8.4 deaths/100,000) and mortality ratio (54%).

**Conclusions:**

In 2019, Asia had an estimated 3.8 million burns comprising nearly half of all the world’s burn cases, deaths, and DALYs. While Asia’s burn indicators have declined since 1990, global improvements have surpassed Asia’s. South and Southeast Asia accounted for the greatest burden of burn morbidity and mortality, but Central Asia consistently had the highest rates relative to overall population. Men were more affected than women, except in South Asia, and the extremes of age (< 5 and 70+ years) suffered the greatest rates of disability and death.